# Road-Roller Technique Using Hyaluronidase-Enhanced Local Anaesthesia for Split-Thickness Skin Grafts

**DOI:** 10.7759/cureus.87956

**Published:** 2025-07-14

**Authors:** Pouya Mafi, Andrej Salibi

**Affiliations:** 1 Plastic Surgery, Queen Elizabeth Hospital, Birmingham, GBR; 2 Plastic Surgery, Kat and Co, Birmingham, GBR

**Keywords:** adrenaline, hyaluronidase, local anaesthetic, skin graft, weight-based dosing

## Abstract

Large split-thickness skin grafts (SSGs) can often be harvested under local anaesthesia, but traditional infiltration requires numerous needle passes across the donor area, which can cause significant discomfort and incomplete anaesthesia, not to mention the risk of exceeding weight-based lidocaine limits. We describe the "road-roller" technique: by combining lidocaine (with adrenaline) and hyaluronidase in one solution, injecting subdermally at the proximal edge of the graft donor area to raise a local bleb, and then using a tightly rolled gauze swab to firm-pressure-spread the anaesthetic "like a road roller", the entire donor site becomes uniformly anaesthetised with a single injection. This method improves patient comfort by avoiding multiple injections and provides a uniform block across the graft. We review the preparation of the anaesthetic mixture (including buffering), step-by-step injection and rolling technique, and outcomes. The technique leverages hyaluronidase's extracellular matrix-degrading properties to enhance diffusion and is supported by evidence from other surgical contexts that hyaluronidase-adjuvanted anaesthesia can reduce pain and improve block quality. We also discuss advantages, limitations, and safety considerations, including rare allergic reactions. In our experience, this road-roller technique reliably provides effective anaesthesia for large SSG harvests in local anaesthetic skin cancer resection and reconstruction while not exceeding the safe anaesthetic dose.

## Introduction

Hyaluronidase is an enzyme that degrades hyaluronic acid in the extracellular matrix, increasing tissue permeability and enhancing the diffusion of injected solutions [1,2]. In plastic and reconstructive surgery, hyaluronidase has long been used as an adjunct to local anaesthetic infiltration. For example, it has been applied in ophthalmic regional blocks and obstetric and gynaecologic anaesthesia to speed the onset and improve the spread of anaesthesia [1]. More recently, its use has expanded into dermatologic and aesthetic procedures. A 2021 review by Sharma and Lahiri notes that hyaluronidase is employed in skin infiltration and split-thickness skin graft (SSG) harvesting, among other plastic surgery applications [2]. By temporarily breaking down the connective tissue cement, hyaluronidase facilitates more uniform anaesthesia over a field. Split-thickness graft donor sites, especially for large wounds, traditionally require many local anaesthetic injections across the marked area. Each needle stick can cause pain, and areas far from the injection points may remain inadequately anaesthetised. Multiple injections increase patient distress and can lead to tissue distortion from fluid spread. In contrast, a single-pass technique has been described to overcome this: by creating a subdermal mound of anaesthetic and then manually distributing it, one can achieve uniform coverage with minimal needle punctures. We have adopted a variant of this, dubbed the road-roller technique, using hyaluronidase in the anaesthetic solution to enhance its spread. This report details our updated protocol for the road-roller single-injection method for large SSG donor sites, including preparation of the anaesthetic mixture, injection steps, and outcome considerations.

## Technical report

**Figure 1:**
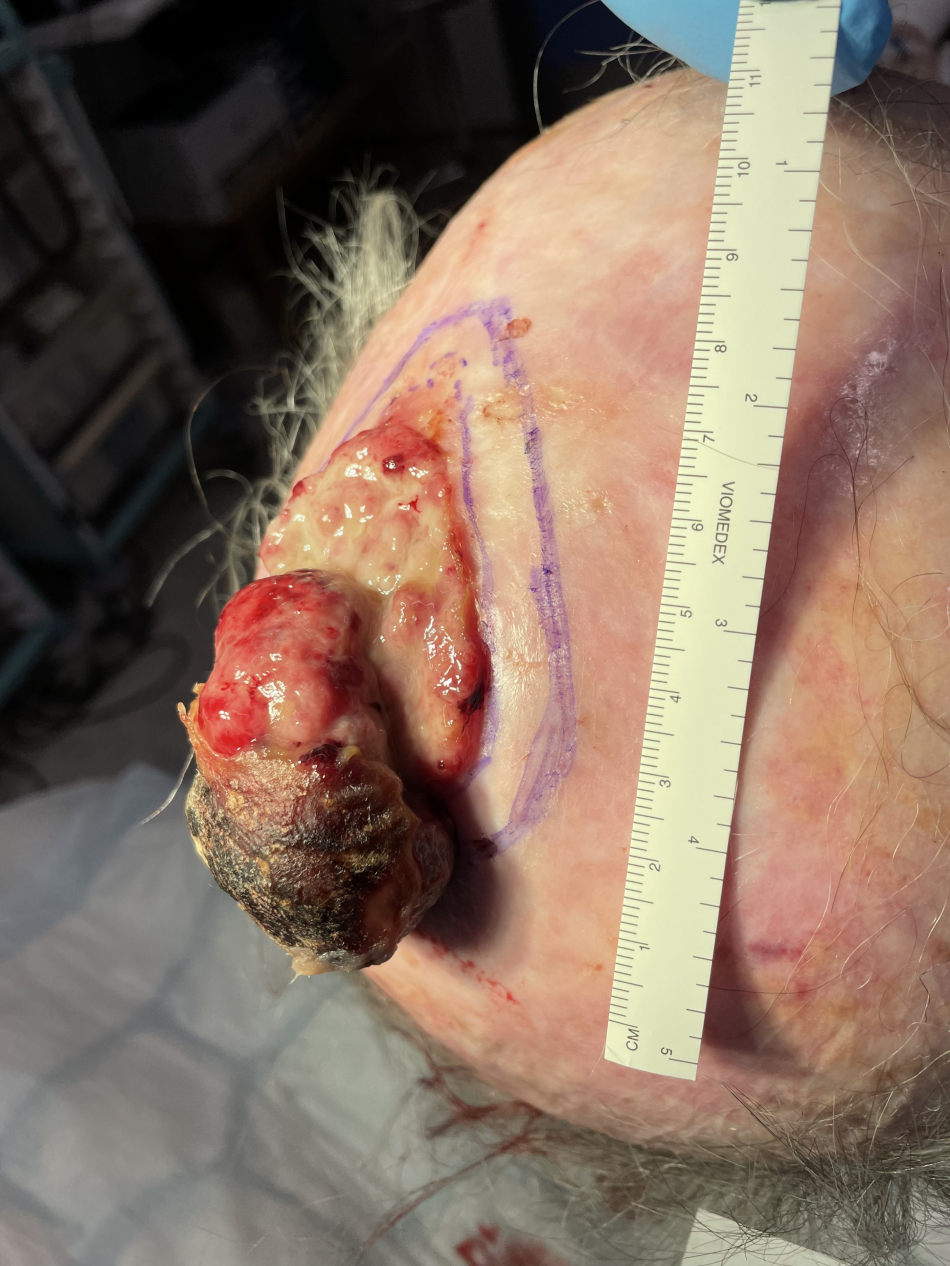
Large fungating scalp lesion

**Figure 2:**
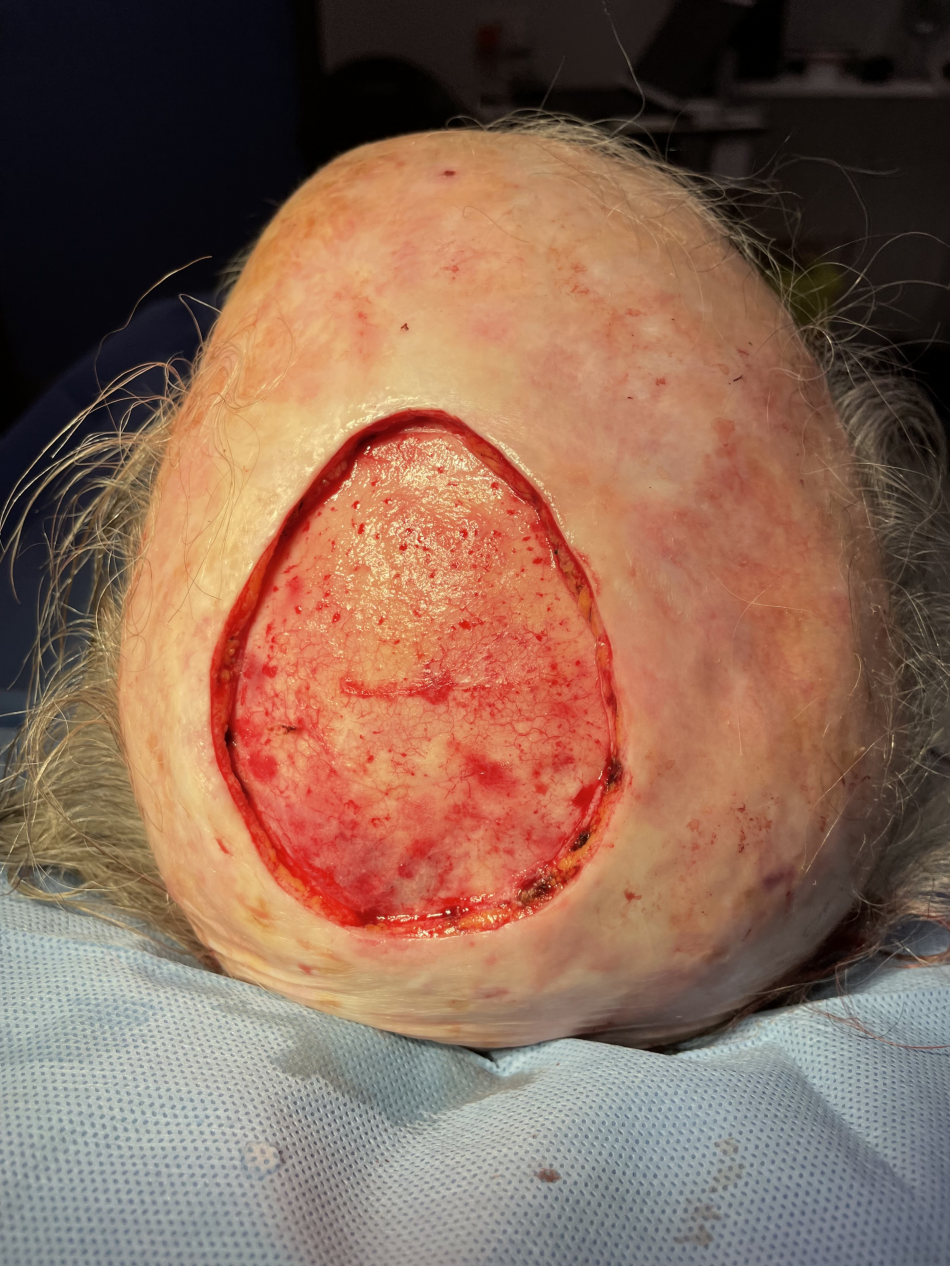
Excised large fungating scalp lesion leaving a large defect

In this technical report, we have demonstrated the road-roller technique following the excision of a large fungating scalp tumour (Figures 1-2).

Preparation

Obtain a mixture of lidocaine 1% with adrenaline (1:200,000) in a 10 mL volume. Buffer this solution with 1 mL of 8.4% sodium bicarbonate to raise pH and reduce injection discomfort. Add one vial of hyaluronidase (1500 units) to the solution and gently mix. The solution is now ready for infiltration.

Initial anaesthesia

**Figure 3:**
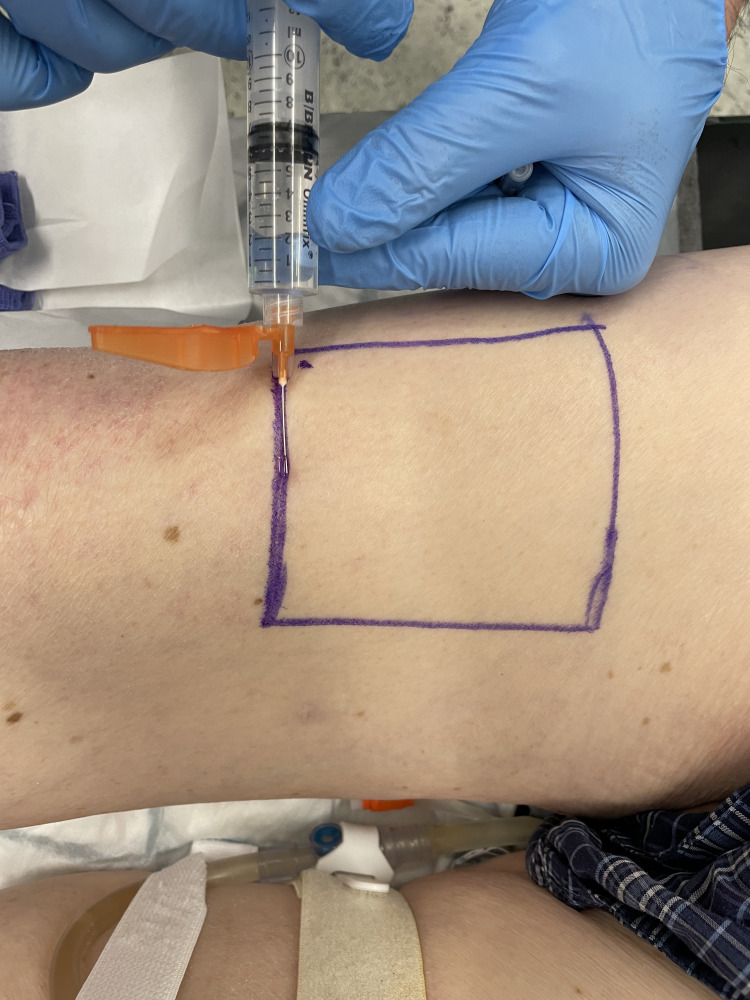
Injection of the local anaesthetic mixture at the proximal edge of the donor area

Under sterile conditions, use a long 27-gauge needle. Insert the needle at the proximal edge of the marked donor site. Inject a portion of the solution subdermally to create a raised bleb or "mound" of local anaesthetic (Figure 3).

Spread along the width

Redirect the needle to run almost parallel to the skin surface and advance slightly. Continue to inject the remaining solution along one half of the donor area's width, essentially covering from the centre to one lateral edge.

Road-roller distribution

**Figure 4:**
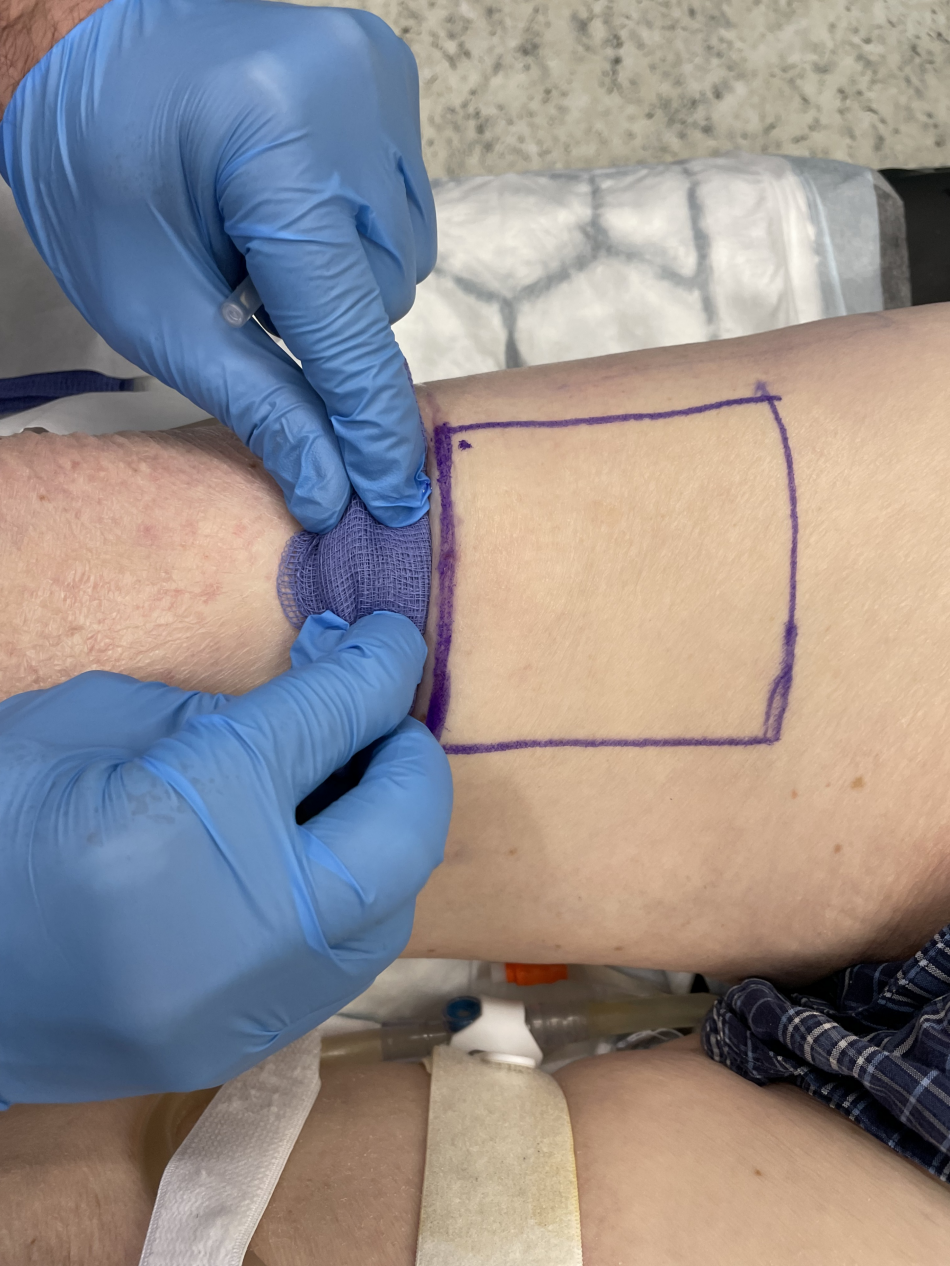
Pressing the gauze firmly just proximal to the injection site

Immediately take a 4×4 gauze swab and tightly roll it into a small cylinder. Place this "roll" at the bleb and press firmly (Figure 4).

**Figure 5:**
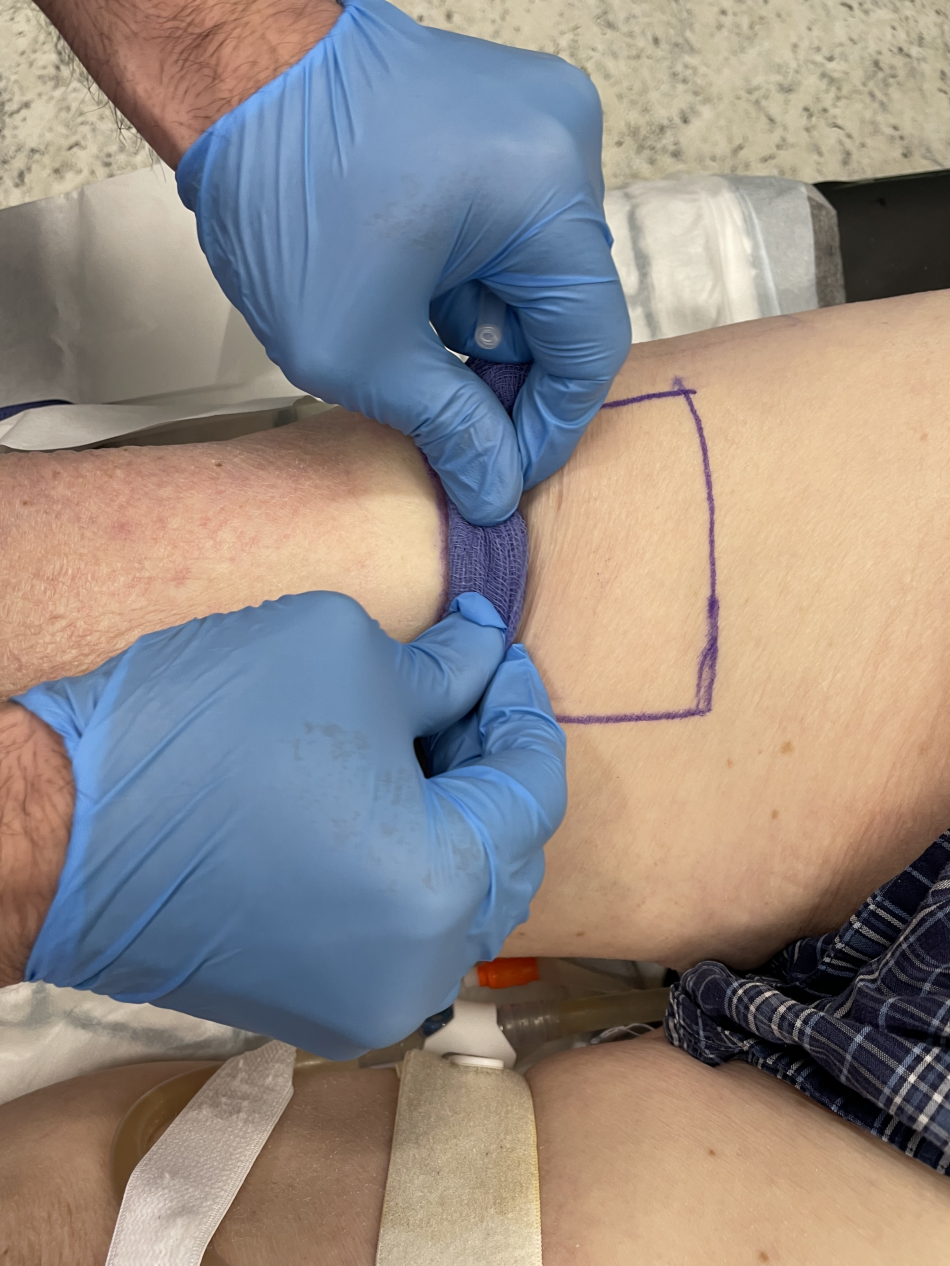
Rolling the gauze across the donor site

**Figure 6:**
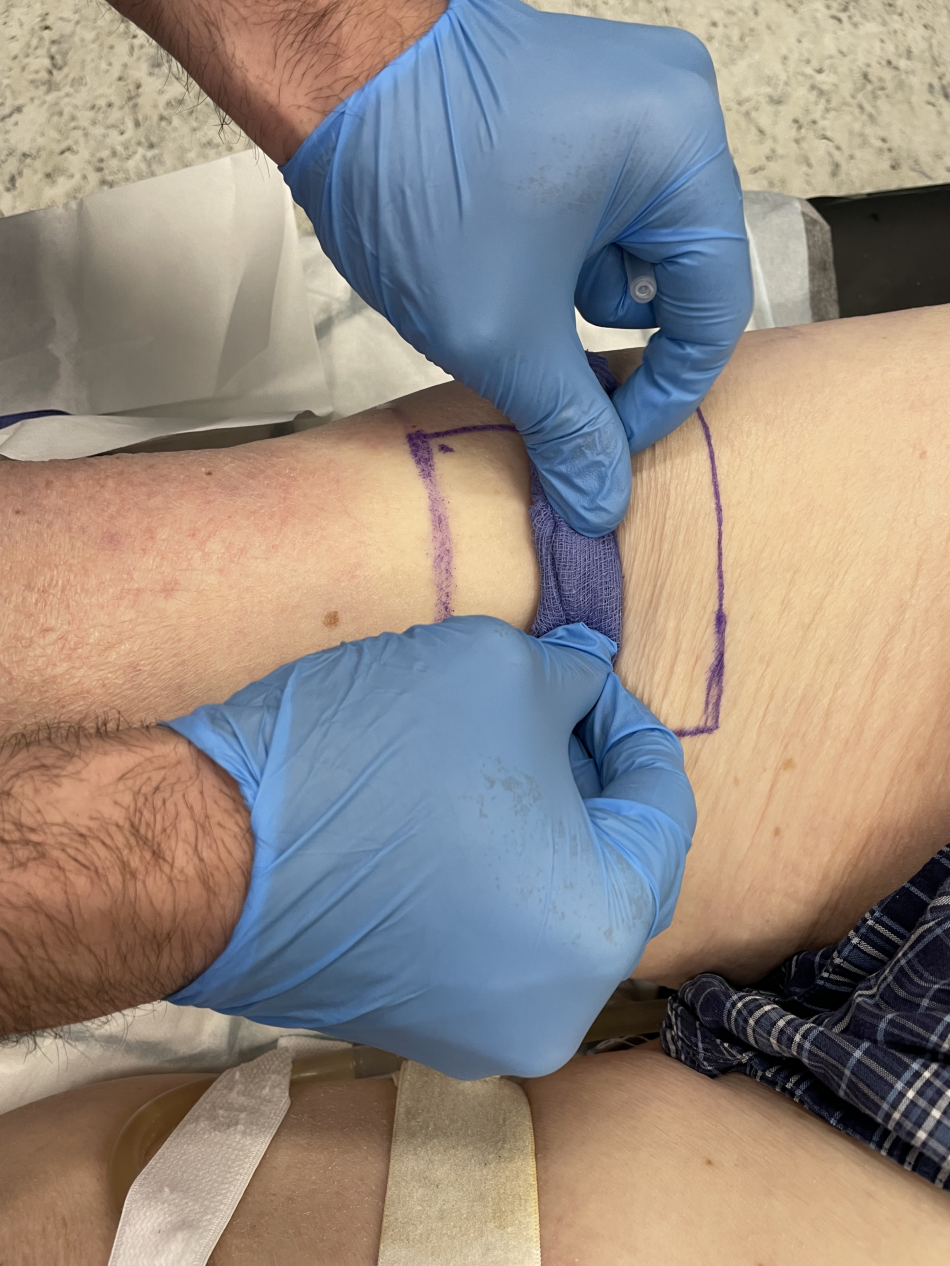
Advancing the gauze further while firm pressure is continuously applied

 Roll or push the swab in straight lines across the graft area, moving it from the already anaesthetised portion distally (Figures 5-6).

The firm pressure ("road-rolling") spreads the subdermal anaesthetic mound along the full length and width of the donor site. Hyaluronidase in the mixture rapidly breaks down tissue barriers, so as you advance the swab, the anaesthetic-hyaluronidase solution diffuses evenly within the same subdermal plane. The skin will blanch due to the adrenaline as you roll the solution through the entire site.

Confirmation

**Figure 7:**
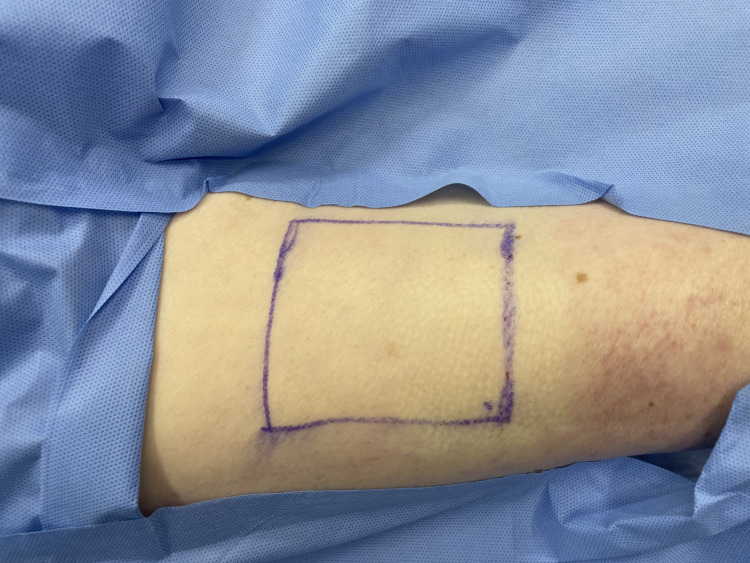
Observing the area for uniform blanching indicating even spread of the local anaesthetic mixture

Observe the donor area for uniform blanching, indicating that the anaesthetic now spans the full site (Figure 7).

Typically, no further needle injections are needed. Wait a few minutes for the full anaesthetic effect. The entire donor site is now uniformly anaesthetised, and a graft can be harvested without additional needle passes. As with any standard method of skin graft harvesting, we recommend that the donor site sensation be checked prior to graft harvest. 

This single-pass road-roller method eliminates the need for multiple injections across the graft area. It uniformly distributes anaesthesia and adrenaline over the donor field, facilitates haemostasis, and aids in harvesting a graft of even thickness. In practice, we have found the technique to be fast and reproducible, with high patient comfort.

## Discussion

The use of hyaluronidase in local anaesthetic infiltrations has shown consistent benefits in various surgeries. In our road-roller technique, hyaluronidase serves as a diffusion spreading factor, enhancing the rate and extent of lidocaine-adrenaline dispersal through the subcutaneous tissue. Clinical studies support these effects: for example, in scalp nerve blocks for craniotomy, the addition of hyaluronidase significantly lowered postoperative pain scores and reduced hemodynamic stress responses without increasing complications [3]. Likewise, a randomised trial in carpal tunnel release found that hyaluronidase-added anaesthetic resulted in significantly less pain after surgery and even reduced operative time [4]. These findings suggest that hyaluronidase can improve the quality and onset of blocks. In the context of SSG harvest, faster spread means more complete anaesthesia for a large area from a single injection.

The road-roller method specifically leverages hyaluronidase and mechanical pressure to produce a uniform field block. By rolling out the injected mound, we avoid patchy areas that can occur with isolated injections. The buffered lidocaine and adrenaline provide a prompt onset and vasoconstriction, while hyaluronidase ensures the mixture does not remain localised. We have found no difficulty in achieving complete anaesthesia of even large thigh donor sites using this single-pass technique.

Importantly, hyaluronidase is generally safe and inexpensive. Reported side effects are few; rare cases of allergic reaction or mild local swelling have been noted. Local administration of hyaluronidase may lead to adverse effects such as localised itching or hypersensitivity, with allergic responses reported in 0.05-0.69% of cases; urticaria and angioedema occur very rarely (less than 0.1%) [5]. In our experience, no unusual adverse events occurred. The addition of adrenaline also provides haemostasis, so donor bleeding is minimised. We did not observe any negative effect on skin graft take.

Potential limitations include the fact that hyaluronidase speeds the wash-in of anaesthetic, which can shorten block duration slightly. However, the anaesthetic duration is typically sufficient to allow for graft harvest and short postoperative pain management. Furthermore, hyaluronidase is ineffective in infected or granulation tissue bed due to fibrin and other altered dense extracellular matrix proteins that form dense physical and chemical barriers; nonetheless, this is not a concern in the donor site. Overall, we find the benefits of rapid, painless coverage far outweigh the drawbacks. Future studies would benefit from the inclusion of patient-reported pain scores comparing standard injection methods to the road-roller technique. 

## Conclusions

The hyaluronidase-enhanced road-roller injection technique offers a simple, effective method for the local anaesthesia of large SSG donor sites. With one needle pass and a single-bolus infiltration and then gentle rolling to spread the solution, a large area becomes uniformly numb, greatly improving patient comfort. This technique avoids multiple painful needle insertions and helps in staying within the safe maximum anaesthetic dose. The use of hyaluronidase amplifies anaesthetic diffusion, and evidence from other surgical contexts supports its efficacy in reducing pain and block failures. We recommend this road-roller method as an adjunctive strategy for dermatologic and reconstructive surgeons harvesting large skin grafts under local anaesthesia.
